# Focal adhesion kinase: predictor of tumour response and risk factor for recurrence after neoadjuvant chemoradiation in rectal cancer

**DOI:** 10.1111/jcmm.12879

**Published:** 2016-05-12

**Authors:** Teresa Gómez del Pulgar, Arancha Cebrián, Maria Jesús Fernández‐Aceñero, Aurea Borrero‐Palacios, Laura del Puerto‐Nevado, Javier Martínez‐Useros, Juan Pablo Marín‐Arango, Cristina Caramés, Ricardo Vega‐Bravo, María Rodríguez‐Remírez, Marlid Cruz‐Ramos, Félix Manzarbeitia, Jesús García‐Foncillas

**Affiliations:** ^1^ Translational Oncology DivisionOncohealth InstituteHealth Research Institute FJD‐UAMUniversity Hospital “Fundacion Jimenez Diaz”MadridSpain; ^2^ Pathology DepartmentOncohealth InstituteHealth Research Institute FJD‐UAMUniversity Hospital “Fundacion Jimenez Diaz”MadridSpain; ^3^ Present address: Pathology DepartmentHospital Clinico San CarlosMadrid28040Spain

**Keywords:** rectal cancer, chemoradiotherapy, focal adhesion kinase, predictive marker, risk factor, neoadjuvant therapy

## Abstract

Rectal cancer represents about 30% of colorectal cancers, being around 50% locally advanced at presentation. Chemoradiation (CRT) followed by total mesorectal excision is the standard of care for these locally advanced stages. However, it is not free of adverse effects and toxicity and the complete pathologic response rate is between 10% and 30%. This makes it extremely important to define factors that can predict response to this therapy. Focal adhesion kinase (FAK) expression has been correlated with worse prognosis in several tumours and its possible involvement in cancer radio‐ and chemosensitivity has been suggested; however, its role in rectal cancer has not been analysed yet. To analyse the association of FAK expression with tumour response to CRT in locally advanced rectal cancer. This study includes 73 patients with locally advanced rectal cancer receiving standard neoadjuvant CRT followed by total mesorectal excision. Focal adhesion kinase protein levels were immunohistochemically analysed in the pre‐treatment biopsies of these patients and correlated with tumour response to CRT and patients survival. Low FAK expression was significantly correlated with local and distant recurrence (*P* = 0.013). Low FAK expression was found to be a predictive marker of tumour response to neoadjuvant therapy (*P* = 0.007) and patients whose tumours did not express FAK showed a strong association with lower disease‐free survival (*P* = 0.01). Focal adhesion kinase expression predicts neoadjuvant CRT response in rectal cancer patients and it is a clinically relevant risk factor for local and distant recurrence.

## Introduction

Rectal cancer represents about 30% of colorectal cancers 1, remaining a significant problem worldwide. In the majority of cases the disease is localized to the primary site with no evidence of distant spread, and in these patients surgical resection currently remains the cornerstone of treatment. Pre‐operative chemoradiation (CRT) and then total mesorectal excision is currently the standard of care for locally advanced stages of rectal cancer, to reduce the probability of recurrence and to possibly improve overall survival 2, 3. The use of neoadjuvant CRT results in a reduction in local recurrence rates when compared to surgery alone 3, 4.

The tumour response to neoadjuvant therapy is assessed as tumour and nodal downstaging and tumour regression grade, which has been correlated with a better outcome 5, 6. While complete response is only observed in 10–30% of cases, in the rest of the cases the residual disease varies from microscopic tumour foci to no response at all 7. Many factors may predict tumour response to CRT 8, 9, 10, 11, 12 but up to now, a model that would predict clinically or pathologically complete or partial tumour response after CRT is not available.

Focal adhesion kinase (FAK) is a cytoplasmic non‐receptor tyrosine kinase that is expressed ubiquitously and specifically localized in focal adhesions 13. This enzyme is involved in the regulation of cell cycle, adhesion, migration and differentiation. The best characterized mechanism that promotes FAK activation involves integrin receptor clustering upon the binding of cells to extracellular matrix proteins. Indirect interactions between FAK and integrins at focal adhesions mediate the integrin‐FAK linkage. In head and neck cancer, the blockade of an integrin‐FAK‐cortactin‐JUN N‐terminal kinase 1 (JNK1) signalling cascade through specific antibodies against β1 integrins renders cells sensitive to radiotherapy and delays xenograft growth 14. It has also been found that overexpression of integrin β1, accompanied by increase in cell adhesion and migration through FAK–AKT signalling, is associated with gefitinib resistance in a non‐small cell lung cancer cell line 15. These results suggest a possible role of FAK in cancer radio‐ and chemosensitivity.

Focal adhesion kinase is a multifunctional regulator of cell signalling within the tumour microenvironment 16, 17, 18, however, some of the functions of FAK in tumorigenesis remain under investigation. Although it has been found that FAK is overexpressed and it correlates with worse prognosis in different tumour types 13, over the last years it has been published that weak expression of FAK is an independent predictor of poor patient outcome in some other tumours 19, 20, 21. The role of FAK in locally advanced rectal cancer with respect to tumour response after CRT or to survival is unclear.

In this study, we performed a single‐centre retrospective analysis of 73 patients treated for locally advanced rectal adenocarcinoma with standard neoadjuvant CRT. Our objective was to investigate FAK expression as a potential predictive marker of preoperative CRT and its correlation with survival.

## Materials and methods

### Patients and treatment

The records of 91 consecutive patients with clinical American Joint Committee on Cancer 22 stage II or stage III rectal adenocarcinoma who underwent standardized pre‐operative CRT followed by total mesorectal excision, from December 2006 to January 2014, were reviewed. Only those patients with available endoscopic biopsies for immunohistochemical analysis were selected for this study (*n* = 73). This study was reviewed and approved by the Institutional Review Board at the University Hospital Fundacion Jimenez Diaz and it was performed according to the REMARK guidelines 23.

Magnetic resonance imaging (MRI), computed tomography, endorectal ultrasound and/or endoscopy were used for staging before CRT in all patients. All the cases were reviewed and, according to the recommendations by the College of American Pathologists, a two‐tiered system was used to grade tumours in two groups: low‐grade (greater than or equal to 50% gland formation) and high‐grade (less than 50% gland formation) 24.

Patients received standard pre‐operative radiotherapy with a dose of 40–50.4 Gy in 1.8–2 Gy/fraction. Concomitant fluoropyrimidines‐based chemotherapy (standard regimen of 5‐FU or capecitabine) was administered. In 14 (19%) of the patients, 5‐FU was combined with oxaliplatin. Follow‐up starts at the beginning of the neoadjuvant therapy. Evaluation of T and N stages was determined after CRT, using MRI. The characteristics of the patients and tumours are summarized in Table 1.

**Table 1:** Clinicopathological characteristics of the patients

Baseline clinical characteristics (*N* = 73)	*N* (%)
Age	
<60	12 (16.4)
>60	61 (83.6)
Gender	
Male	46 (63.0)
Female	27 (37.0)
ECOG Performance status	
0	40 (54.8)
1	31 (42.5)
2	2 (2.7)
Neoadjuvant chemotherapy	
RT + 5‐FU	59 (80.8)
RT + 5‐FU+oxaliplatin	14 (19.2)
Grade differentiation	
Low	57 (78.1)
High	16 (21.9)
Stage	
II	4 (5.5)
III	68 (93.2)
N/A	1 (1.4)
Pathological response	
Responder	36 (49.3)
Non‐responder	37 (50.7)
T‐Downstaging	
No	28 (38.4)
Yes	39 (53.4)
N/A	6 (8.2)
N‐Downstaging	
No	20 (27.4)
Yes	47 (64.4)
N/A	6 (8.2)
Status	
Death	8 (11.0)
Alive with disease	6 (8.2)
Alive without disease	58 (79.5)
Loss of follow‐up	1 (1.4)

ECOG: Eastern Cooperative Oncology Group; RT: radiotherapy; N/A: not available.

### Histological grading of tumour regression

To evaluate regression grade, T‐downstaging and N‐downstaging, all the specimens of rectal resection following neoadjuvant therapy were fixed with formaldehyde for 24 hrs. The complete tumour area was embedded in paraffin blocks and histologically analysed. Serial sections were cut from each block and stained with haematoxylin and eosin. Histological assessment of tumour regression was performed by an experienced pathologist. The regression grade was evaluated in the area with the least response to treatment. The percentage of viable residual tumour cells was estimated according to recommendations settled by the College of American Pathologists. Ryan's criteria were used to quantify response as follows: grade 0 (complete response: absence of tumour cells); grade 1 (moderate response: fibrosis with isolated tumour cells); grade 2 (minimal response: tumour nests outgrown by fibrosis); and grade 3 (poor response: minimal or no tumour kill). T‐downstaging was considered to be any reduction in the pathologic T stage *versus* pre‐treatment stage. N‐downstaging was defined as post‐operative N stage lower than pre‐radiotherapy clinical N stage.

### Immunohistochemical evaluation

Formalin‐fixed paraffin‐embedded (FFPE) tissue samples obtained by punch biopsy before pre‐operative CRT were used for Tissue Microarray (TMA) construction. Representative tumour regions from biopsies were identified by a pathologist on haematoxylin and eosin‐stained tissue sections. After pathologist review, TMAs were assembled from triplicate 0.6 mm cores of FFPE biopsy tumour samples using a TMA workstation MTA‐1 (Beecher Instruments, Sun Prairie, WI, USA).

All the immunohistochemical techniques were performed in the Pathology Department at Fundacion Jimenez Diaz. The immunohistochemistry procedure was performed with a Dako Autostainer (Dako, Glostrup, Denmark). The sections were stained using the rabbit polyclonal anti‐FAK (1:50; Cell Signaling, Danvers, MA, USA) which recognizes total FAK protein. Immunohistochemistry was evaluated by two independent pathologists with expertise in gastrointestinal pathology. Optic microscopes were used to evaluate both the percentage of stained cells and also the intensity of staining with a semiquantitative scale in three grades. To asses reliability we measured interobserver agreement with over 90% concordance. Cases with discordant results were reviewed co‐jointly to reach consensus. Both pathologists were blinded to the outcome of the patients.

### Statistical analysis

Correlation between FAK expression and clinicopathological variables or response to neoadjuvant therapy was evaluated by Mann–Whitney test. The relationship between FAK expression and each clinical variable with the pathological response was assessed using binary logistic regression model in both univariate and multivariate.

To assess the association of FAK expression with survival, expression *versus* no expression was used as a cut‐off point. Disease‐free survival was defined as any relapse from the time of surgical procedure. Overall survival was calculated from the time of surgical procedure to the date of death or last follow‐up. Survival analysis was assessed by using the Kaplan–Meier method, with log‐rank test assessing the differences between the groups of FAK expression. Cox regression model was conducted to assess the hazard ratio. Multivariate analysis was performed adjusting for known confounders as age, stage, grade of differentiation and adjuvant treatment. Values of *P* < 0.05 were considered statistically significant. All reported *P*‐values were two‐sided. The SPSS software version 20.0 (IBM company, Armonk, NY, USA) was used for all the statistical analyses.

## Results

Ninety‐one consecutive rectal cancer patients were reviewed. Endoscopic biopsies before CRT were available for 73 patients (46 men and 27 women) that were finally included in the study. The median age was 74 years (range 48–90). Evaluation of tumour response to pre‐operative CRT showed that 9 patients (12%) had a complete response and 27 (36%) had only isolated tumour cells, all these patients were considered as responders. Patients with minimal and poor response, 15 (21%) and 22 (30%), respectively, were grouped as non‐responders. Thirty‐nine patients (53%) and 47 patients (64%) had T‐ and N‐downstaging, respectively, after CRT. After surgery, 6 of 73 patients developed a local recurrence and 13 patients developed distant metastasis.

### Correlation between FAK expression and clinicopathological parameters

**Figure 1:**
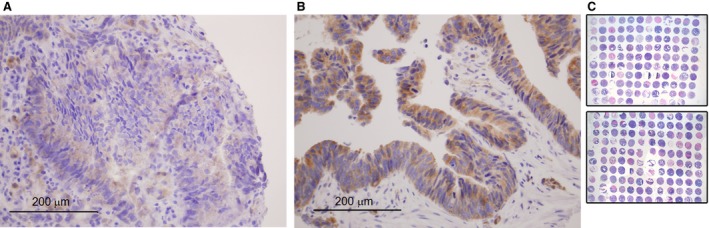
Representative pictures of FAK expression and whole Tissue Microarrays. Immunohistochemical analysis showing weak (**A**) and strong (**B**) specific cytoplasmic expression of FAK in rectal cancer biopsies before CRT. (**C**) Haematoxylin and eosin staining of triplicate cores of all biopsy tumour samples used for the assessment of FAK expression.

**Figure 2:**
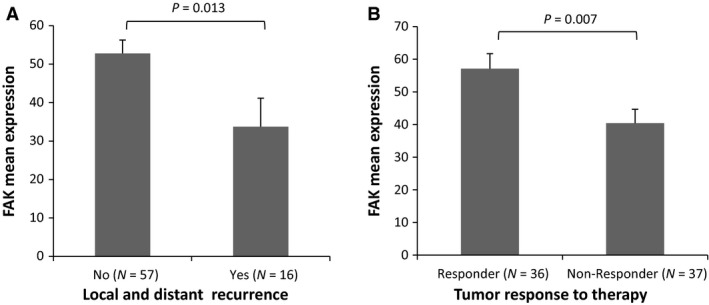
Boxplots show distribution of FAK expression according to local and distant recurrence (**A**), and tumour response to therapy (**B**). Significant changes in mean expression for FAK between subgroups were calculated by Mann–Whitney test and indicated in the boxplot.

Immunohistochemical analysis in tissue samples collected before CRT was performed to determine the FAK expression level. A human bladder tissue was used as a positive control for immunohistochemical staining for FAK (according to the human protein atlas at http://www.proteinatlas.org). Figure 1 shows the cytosolic localization of FAK and results of immunohistochemical staining revealed that median percentage of cells staining positive for FAK was 60 (range 0–90). Eight of 73 patients (11%) were negative for staining and the rest of cases (89%) showed a wide range of FAK expression. There were no significant differences in FAK expression levels with respect to age, gender, ECOG performance status, TNM stage and grade of differentiation (Table 2). However, a significant correlation was found between low FAK expression and the incidence of local and distant metastases (*P* = 0.013; Fig. 2A, Table 2) since 5 of the 16 patients (31%) with recurrence had no expression of FAK compared to 3 of the 57 patients (5%) without any recurrence.

**Table 2:** Correlation of FAK and clinicopathological parameters

Variable	Median	Interquartile range	*P*
Age			
<60	65	53	0.600
>60	50	45	
Gender			
Male	55	51	0.908
Female	60	50	
ECOG performance status			
0	55	58	0.953
1	60	40	
Stage			
II	30	18	0.141
III	60	45	
Grade of differentiation			
Low	60	48	0.191
High	35	54	
T‐Downstaging			
No	45	39	0.169
Yes	60	50	
N‐Downstaging			
No	45	34	0.297
Yes	60	50	
Local and distant recurrence			
No	60	50	**0.013**
Yes	25	60	
Pathological response			
No	40	40	**0.007**
Yes	65	44	

ECOG: Eastern Cooperative Oncology Group. Bold *P* values are significant at the 0.05 level.

### Tumour response and survival analysis

No significant correlations between FAK protein levels and T‐downstaging or N‐downstaging were observed (Table 2). Nevertheless, FAK expression levels were significantly associated with the histopathological tumour response to CRT, the mean expression was lower in non‐responder patients than responders (*P* = 0.007; Fig. 2B, Table 2). Binary logistic regression model was performed to assess FAK expression as predictor of response. Univariate analysis showed an association with response (OR: 1.02, 95% CI: 1.00–1.04, *P* = 0.012), that was maintained as significant after adjustment for potential confounders, age and gender, suggesting FAK as an independent predictor of pathological response.

**Figure 3:**
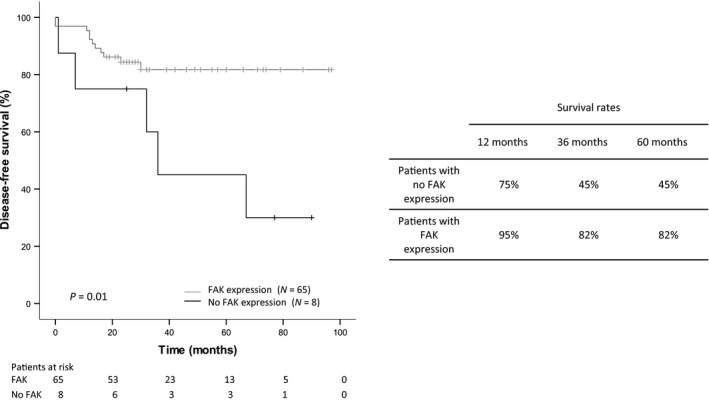
Kaplan–Meier survival analyses of rectal cancer patients receiving neoadjuvant CRT treatment according to FAK expression levels in pre‐treatment biopsies. Patients in the FAK expression group showed significantly better disease‐free survival than those in the no expression group. Data are based on immunohistochemical results of pre‐treatment biopsies.

With a median follow‐up of 32 months (range 7–97 months), 16 of 73 patients developed local recurrence and/or distant metastasis. No expression of FAK was found to be significantly associated with reduced disease‐free survival (*P* = 0.01 by the log‐rank test, Fig. 3; HR: 3.56; 95% CI: 1.22–10.38, *P* = 0.02). The 1‐, 3‐ and 5‐year survival rates for these patients were 75%, 45% and 45% respectively. The median survival time was 36 months. Patients whose tumours expressed FAK had 1‐, 3‐ and 5‐year survival rates of 95%, 82% and 82% respectively and the median survival time was not reached. A multivariate analysis was conducted using the confounding variables age, stage, grade of differentiation and adjuvant treatment as covariates. FAK expression remained as an independent prognostic marker of lower risk of recurrence (HR: 4.56; 95% CI: 1.44–14.45, *P* = 0.010; Table S1). Overall survival was also assessed but it did not meet statistical significance (*P* = 0.254).

## Discussion

Pre‐operative CRT is the standard of care for patients with locally advanced rectal cancer 2, 3. After this treatment a complete tumour response is observed in 10–30% of cases 7, since all patients do not have the same sensitivity to CRT. Analysis of predictive factors of response to this pre‐operative treatment could help physicians to distinguish between patients that are therefore more likely to respond well from those unlikely to do so.

The role of FAK in radiation sensitivity has been poorly studied and it seems to be controversial. There is a report showing that overexpression of FAK in HL‐60 cells confers marked resistance 25 and another in which siRNA‐mediated FAK knock‐down promotes radio‐sensitization in pancreatic cancer cells 26. However, a study on advanced squamous cancer cells showed that the absence of FAK can release constrained FAK places on signalling from p53 to the induction of several target genes, namely p21 and a subset of genes involved in DNA repair. Moreover, cells without FAK appear to be more efficient at repair after radiation‐induced DNA damage. Thus, FAK functions to suppress the radiation‐induced DNA repair by blocking induction of p21, consequently loss of FAK is linked to enhanced resistance to ionizing radiation 27. Our results are in accordance with this study since we observed that low FAK levels were significantly associated with the absence of response (minimal and poor response) to neoadjuvant CRT treatment supporting that FAK loss induces radio‐resistance in rectal cancer patients and it could be a potential predictive marker. Considering the increasing evidence that the treatment of rectal cancer must be individualized, if this biomarker is validated, it might be used for stratifying patients and included in models for prediction, and therefore adapts the treatment to the patient.

On the other hand, rectal cancer behaves differently from colon cancer, particularly with respect to an increased risk for local recurrence because of the differences in the lymphatic drainage and the narrow anatomic space of the pelvis. Although the reduction in the rate of local recurrence is higher after pre‐operative radiotherapy, when compared to post‐operative radiation alone in primary local advanced rectal cancer cases, about 20% patients will suffer recurrence or distant metastasis 28. It has been found that risk factors associated with local recurrence in patients with rectal cancer receiving neoadjuvant therapy are different from the traditional factors in patients treated with surgery and/or adjuvant therapy alone 29.

Focal adhesion kinase is typically located at structures known as focal adhesions from where it transduces signals into cells that control multiple cancer‐related events, including migration, invasion, angiogenesis, protection of cells from suspension‐induced cell death and proliferation in three‐dimensional matrices 30, 31, 32, 33, 34.

In this study, we observed that no FAK expression was significantly associated with a shorter DFS, suggesting FAK as a potential risk factor. This result would be supported by molecular studies which describe an increased carcinoma cell migration after inhibition of FAK in HeLa cervical cancer cells 35 and a negative role for FAK during the invasion of different types of carcinoma cells 36. These studies show that the down‐regulation of FAK is required and sufficient for detachment of cells from the extracellular matrix, leading to increased tumour cell motility, invasion and metastasis. The detached cells migrate to a new site, followed by reactivation of FAK and reattachment to the extracellular matrix, developing metastatic deposits. It is also supported by experiments using FAK knockdown cells which exhibited remarkably enhanced motility after detachment and migrated out from the cells sheet in wound‐healing assay. The down‐regulation of FAK is essential for early metastatic spreading, enabling vascular circulation of tumour cells without adhesion. Following this, rectal cancer patients with no FAK expression in the biopsy before pre‐operative CRT are more likely to have detached cells responsible for future metastases than those patients expressing FAK. It is expected that these patients have higher incidence of recurrence and worse disease‐free survival as we observed in our work.

This is the first report describing the impact of FAK expression in rectal cancer patients as a predictive and prognostic marker. The association of low FAK levels with the outcome in these patients is an unexpected finding since most of the previous studies in different tumour types point high levels of FAK as a prognostic biomarker and potential therapeutic target 13. However, recent studies have described that weak expression of FAK is a strong independent predictor of poorer disease outcome. In human cervical cancer, patients with low expression of FAK were characterized by a significantly poor overall survival 20. Interestingly, these authors also found a significant inverse correlation between FAK expression and pelvic lymph node metastasis, recurrent disease and survival status. These observations are in accordance to our results in rectal cancer. Reduced expression of FAK has also been associated with poor survival in intrahepatic cholangiocarcinoma 37, extrahepatic bile duct carcinoma 38 and ovarian cancer 39. The association of low FAK levels and outcome has also been observed in haematological malignancies. Focal adhesion kinase expression was an independent prognostic factor and showed a tendency to be associated with tumour response to therapy in diffuse large B‐cell lymphoma 19. In chronic lymphocytic leukaemia, increased FAK expression was associated with improved outcome in patients treated with rituximab, fludarabine and cyclophosphamide immunochemotherapy 21. All together suggests that FAK might play different roles in different tumour types or during different stages of tumour progression.

So far, most of the studies have suggested that FAK functions drive various tumour‐promoting signalling pathways, and small‐molecule FAK inhibitors are emerging as promising chemotherapeutics which are being evaluated in different clinical trials 13. However, it is important to record that FAK's role in cellular responses to ionizing radiation, and pro‐survival signalling in general, may be context dependent, and that there needs to be caution when considering therapeutic combinations of FAK inhibitors and radiotherapy, as this may not always be clinically beneficial.

One of the limitations to our study that warrant consideration is that it has been performed in a limited cohort. To address whether FAK expression could be a suitable marker for identifying patients at higher risk in rectal cancer, we are continuing to collect specimens from additional patients to validate our results as well as extending our studies to an independent patient cohort.

In conclusion, we have identified low FAK expression associated with tumour resistance to neoadjuvant CRT and worse disease‐free survival in rectal cancer patients. This is an important finding because it helps to identify a subset of patients with rectal cancer who will most likely not respond to CRT.

## Conflict of interest

The authors confirm that there are no conflicts of interest.

## Author contribution

TGP and AC designed the study, analysed the data and wrote the paper; MJFA interpreted the immunohistochemical results and analysed the data; ABP, JMU, RVB and MRR performed the research; LPN generated the clinical database and analysed the data; JPMA, CC, and MCR reviewed the records of rectal cancer patients^;^ FM interpreted the Immunohistochemical results; JGF designed the study and revised the paper critically.

## Supporting information


**Table S1** Univariate and multivariate analyses of FAK expression and disease‐free survival in rectal cancer patients.
