# Supplementary material for: Focal adhesion kinase: predictor of tumour response and risk factor for recurrence after neoadjuvant chemoradiation in rectal cancer
Source: J Cell Mol Med. 2016 May 12;20(9):1729–36. doi: 10.1111/jcmm.12879 (PMC4988282; doi:10.1111/jcmm.12879)
Supplement: Supplementary file 1 — Table S1 Univariate and multivariate analyses of FAK expression and disease‐free survival in rectal cancer patients. [file JCMM-20-1729-s001.docx]

| Variables | Univariate analysis | Multivariate analysis | | |  |  |  |
| --- | --- | --- | --- | --- | --- | --- | --- |
|  | **HR (95% CI) *P*** | | **HR (95% CI) *P*** | | | |  |
| FAK  Expression vs.  No expression  Age  Stage  III vs. II  Grade of differentiation  High vs. Low  Adjuvant treatment  Yes vs. No | 3.56 (1.22- 10.38) 0.020  0.97 (0.92-1.01) 0.128  0.75 (0.10-5.76) 0.784  1.13 (0.36- 3.52) 0.832  0.84 (0.24-2.94) 0.779 | | | 4.56 (1.44-14.45) 0.010  0.95 (0.90-1.01) 0.078  0.63 (0.08-5.03) 0.664  0.84 (0.26-2.77) 0.779    0.49 (0.12-2.02) 0.321 |  |  |  |

**Supplementary Table 1.** Univariate and multivariate analyses of FAK expression and disease-free survival in rectal cancer patients.
